# Outcomes after transcatheter aortic valve replacement in cancer survivors with prior chest radiation therapy: a systematic review and meta-analysis

**DOI:** 10.1186/s40959-020-00062-y

**Published:** 2020-07-14

**Authors:** Meer Rabeel Zafar, Syed Farrukh Mustafa, Timothy W. Miller, Talal Alkhawlani, Umesh C. Sharma

**Affiliations:** 1 Department of Medicine, Division Cardiology, Jacob’s School of Medicine and Biomedical Sciences, 875 Ellicott Street, Suite 7030, Buffalo, New York, 14203 USA; 2 Department of Internal Medicine, William Beaumont Hospital, Royal Oak, MI USA

**Keywords:** Radiation, Cancer Survivior, TAVR, Meta-analysis

## Abstract

**Background:**

Cancer survivors with prior chest radiation therapy (C-XRT) frequently present with aortic stenosis (AS) as the first manifestation of radiation-induced heart disease. They are considered high-risk for surgical valve replacement. Transcatheter aortic valve replacement (TAVR) is as an attractive option for this patient population but the outcomes are not well established in major clinical trials. The authors performed a systemic review and meta-analysis of clinical studies for the outcomes after TAVR in cancer survivors with prior C-XRT.

**Methods:**

Online databases were searched from inception to April 2020 for studies evaluating the outcomes of TAVR in patients with and without C-XRT. We analyzed the pooled estimates (with their 95% confidence intervals) of the odds ratio (OR) for the all-cause mortality at 30-day and 1-year follow-ups, 4-point safety outcomes (stroke, major bleed, access-related vascular complications and need for a pacemaker), a 2-point efficacy outcome (mean aortic valve gradient and left ventricular ejection fraction) and worsening of congestive heart failure (CHF). Four studies were included following 2054 patients with and without prior C-XRT exposure (164 patients and 1890 patients respectively).

**Results:**

The C-XRT group had similar 30-day mortality compared to the control group (OR 1.29, 95% CI 0.64 to 2.58, *p* = 0.48). The 1-year mortality was higher in the C-XRT group (OR 1.97, CI 1.15 to 3.39, *p* = 0.01). Apart from higher congestive heart failure (CHF) exacerbation in the C-XRT group (OR 2.03, CI 1.36 to 3.04, *p* = 0.0006), TAVR resulted in similar safety and efficacy outcomes in both groups.

**Conclusion:**

TAVR in the C-XRT group has similar 30-day mortality, safety, and efficacy outcomes compared to the control group; however, they have higher 1-year mortality and CHF exacerbation. Including an oncologist to the cardiology team who considers cancer stage in the decision-making process and applying additional preoperative scores such as frailty indices may refine the risk assessment for these patients. The quality of analyzed data is modest, warranting randomized trials to assess the true benefits of TAVR in these patients.

## Introduction

Valvular heart disease occurs in approximately 81% of cancer survivors with prior chest radiation therapy (C-XRT) and a frequent initial manifestation is aortic-valve stenosis (AS) [1]. With increased longevity, a substantial portion of these patients develop symptomatic AS [2–4]. The mortality rate is up to 90% in a 2-year natural history of patients having symptomatic AS [5, 6]. The European Society of Cardiology position paper on cardiovascular toxicity related to cancer therapeutics recommends using angiotensin-converting enzyme inhibitors or angiotensin II receptor blockers for afterload reduction to attenuate heart failure induced by chemo-radiation therapies [7]. In patients with severe AS, effective afterload reduction is mainly achievable by aortic valve intervention. In cancer survivors with prior C-XRT and severe AS, transcatheter aortic valve replacement (TAVR) has been suggested as a safer modality as compared to surgical aortic valve replacement (SAVR), since mediastinal fibrosis and aortic calcification that happen after radiation makes open-heart surgery quite challenging [8].

Desai and colleagues recently reported significantly worse short and long-term survival in cancer patients with prior C-XRT who underwent SAVR [8]. TAVR is currently an accepted intervention for symptomatic AS, regardless of surgical risk [9]. However, there is limited data for TAVR in cancer survivors as outcomes in this patient poulation were not adequately assessed in major clinical trials. Consequently, these patients are left with equivocal treatment options, and the heart team is compelled to offer treatment choices based on the assumption of cancer prognosis and quality of life.

A recent observational study from our group found significantly increased long-term mortality among cancer survivors with C-XRT undergoing TAVR [10]. Increased mortality in these patients may be attributed to latent effects of cancer progression and therapeutic regimens, including C-XRT. Since there is a lack of standardized guidelines for management, we performed a systemic review and meta-analysis of the available studies to evaluate mortality, safety, and efficacy outcomes for TAVR in this challenging cohort. To the best of our knowledge, this is the first meta-analysis addressing this clinical question with the main objective to reaffirm the findings from individual studies and providing clinicians a chance to make better-informed decisions.

## Methods

This study was conducted according to the recommendations of the Preferred Reporting Items for Systemic Reviews and Meta-Analysis [11]. No ethics committee approval was required because we performed a meta-analysis of already published studies in the literature.

### Search strategy

Two investigators (M.R.Z. and S.F.M.) independently searched MEDLINE, PubMed, Google Scholar, and Cochrane databases from inception to April 2020. The following keywords were used: transcatheter aortic valve implantation, transcatheter aortic valve replacement, aortic stenosis, malignancy, cancer survivors and radiation therapy. Potentially relevant citations were retrieved from reference lists of the identified reports and relevant reviews.

### Study selection and eligibility criteria

After the identification of all relevant studies, 2 authors (M.R.Z. and S.F.M.) independently performed study selection, and discrepancies were resolved by consensus and arbitration by the senior author (U.C.S). Eligible studies met the following criteria: 1) Population: Patients with a history of thoracic malignancy and severe AS; 2) Exposure: C-XRT; 3) Control: Patients with severe AS but without C-XRT; 5) Intervention: TAVR; 6) Main outcomes: All-cause mortality at 30-day and 1-year follow-ups; 7) Additional outcomes: The post-procedural 4-point safety outcomes (stroke, major bleed, access-related vascular complications and need for a pacemaker), a 2-point efficacy outcomes (post-procedural mean aortic valve gradient and left ventricular ejection fraction) and worsening of the congestive heart failure. The safety outcomes were analyzed at 30-day follow up according to Valve Academic Research Consortium-2 definitions [12]; 8) Study design: Published randomized and non-randomized (prospective and retrospective observational) studies. We excluded studies there were not reported in the English language.

### Risk of bias assessment

The risk of bias in the included studies was evaluated independently by two investigators (M.R.Z. and S.F.M.) using the ‘Newcastle-Ottawa Scale’ assessment tool, which assesses the selection, comparability, and outcome assessment biases [13]. The investigators assessed the risk of bias for the included studies and assigned a score for each category.

### Data synthesis and statistical analysis

The data supporting this meta-analysis are from reported studies, which have been cited. The statistical analysis was performed by using the software Review Manager (RevMan Version 5.3). We used the Mantel-Haenszel (MH) method for each clinical outcome and pooled estimates of odds ratio (OR) with 95% confidence interval (CI) and p valves were considered statistically significant at less than 0.05. In some studies, the long term mortality was reported in the graphical form (Kaplan-Meier survival curve). All-cause mortality at 1-year follow-up was extracted from the survival curve graphs by using the percentage formula. For changes in the residual mean gradient and ejection fraction outcomes, an analysis was done using the inverse variance method to calculate the mean difference. A random-effects model was employed as it considers the variability among studies [14]. We applied the I^2^ index and X^2^*p* value (using Cochran’s Q test) to examine heterogeneity among the included studies. The extent of heterogeneity among studies using the I^2^ index was interpreted as follows: 0, 25, 50, and 75% represent zero, low, moderate, and high heterogeneity, respectively [15]. Forest plots were generated to show the relative effect size of the comparison groups for each clinical outcome. To assess publication bias we prepared funnel plots, reported in the Additional file 1.

## Results

### Literature search results

**Fig. 1:**
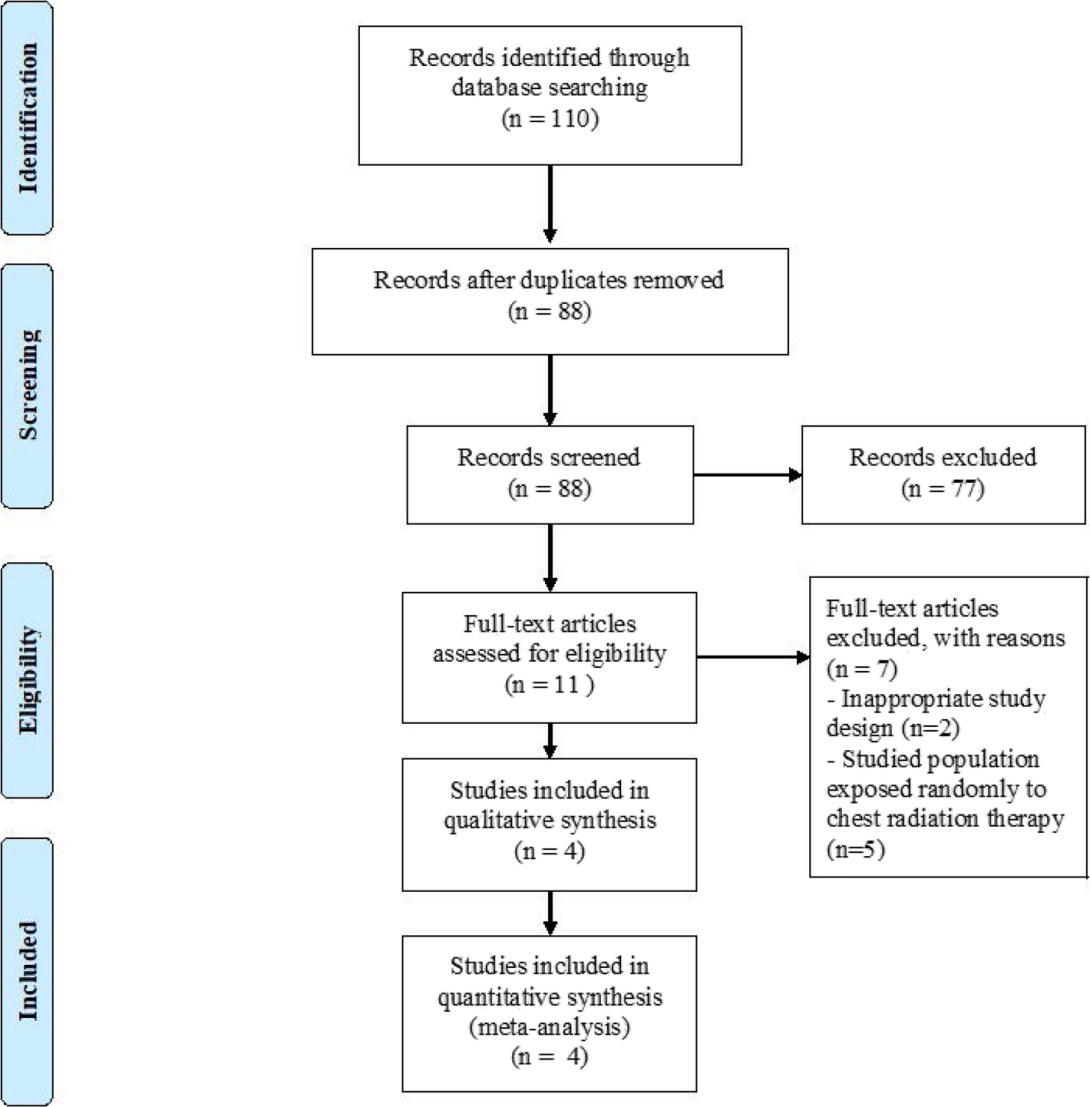
Preferred Reporting Items for Systematic Reviews and Meta-Analyses (PRISMA) flow chart of the studies evaluated

A total of 110 potentially relevant citations were identified and screened from the initial search. After the removal of duplicated studies, we retrieved 11 full-text articles for evaluation of which 4 observational studies satisfied our selection criteria [10, 16–18]. Preferred Reporting Items for Systemic Reviews and Meta-Analyses (PRISMA) flow chart of the study selection is shown in Fig. 1. Major excluded articles [19–25] with reasons are reported in the Additional file 1. The 4 included studies enrolled a total of 2054 patients; 164 patients with prior C-XRT, and 1890 patients without prior C-XRT. The summary of the included studies and their main findings are shown in Table 1 and the baseline characteristics of their population are shown in Table 2.

**Table 1:** Summary of included studies

Study ID	Design	Population	Follow-up	Main findings
Dijos et al. [16]	Single center, prospective cohort study	198 patients with severe AS (19 with prior C-XRT)	6 months	Similar short-and mid-term mortalities between the comparison groups
Bouleti et al. [17]	Single center, prospective cohort study	52 patients with severe AS (26 with prior C-XRT)	5 years	Trends for higher short- and long-term mortalities in C-XRT group, but statistically not significant
Gajanana et al. [18]	Single center, prospective cohort study	1150 patients with severe AS (44 with prior C-XRT)	1 year	Similar short-term mortality in both groups, but higher 1-year mortality in C-XRT group
Agrawal et al. [10]	Observational study (STS/ACC TVT Registry)	610 patients with severe AS (75 with prior C-XRT)	17 months	Significantly higher in-hospital and long-term mortalities in the C-XRT group

STS/ACC TVT Registry- American College of Cardiology National Cardiovascular Data Registry; AS-aortic stenosis; C-XRT- prior chest radiation therapy

**Table 2:** Baseline characteristics of the included studies

	Groups	Dijos [16]	Bouleti [17]	Gajanana [18]	Agrawal [10]
**Demographics**					
Age	C-XRT	68.3 ± 1.7*	73.4 (61.3–83.6)	76 ± 13*	81.64 ± 7.81
	Control	82.5 ± 6.6	73.3 (67.8–83.1)	82 ± 8	82.67 ± 7.98
Male sex	C-XRT	7 (36.84)	13 (50)	10 (23) *	29 (38.66)
	Control	101 (56.4)	13 (50)	583 (51)	291 (54.39)
BMI ((kg/m^2^)	C-XRT	25.9 ± 5.1	21.9 (18.7–24.9)*	29.1 ± 8.9	27.14 ± 6.32
	Control	27.1 ± 5.7	27.9 (22.9–29.8)	28.2 ± 8.6	28.11 ± 5.98
**Comorbid conditions**					
Hypertension	C-XRT	9 (47.31) *	12 (46)	37 (86)	66 (88)
	Control	139 (77.6)	22 (85)	1062 (93)	476 (88.9)
Diabetes mellitus	C-XRT	1 (5.3) *	0 (0)	13 (31)	31 (41.3
	Control	56 (31.3)	7 (2)	392 (34)	176 (32.5)
Coronary Artery disease	C-XRT	9 (47.3)	14 (54)	3 (7)	50 (66.67)
	Control	104 (58.1)	12 (46)	193 (17)	307 (57.3)
Prior stroke	C-XRT	0 (0)	1 (4)	4 (9)	10 (13.33)
	Control	11 (6.1)	2 (8)	125 (12)	53 (9.9)
**Risk scores and Echocardiographic characteristics**					
STS score (%)	C-XRT	NR	5.0 (2.9–6.1)	7 ± 4	8.1 (5.1–11)
	Control		4.7 (3.0–8.7)	8 ± 5	8.1 (5.3–11)
LVEF (%)	C-XRT	57 ± 11.3	60 (45–60)	53 ± 11	55.65 ± 12.40
	Control	53.8 ± 14.8	60 (45–60)	52 ± 13	54.46 ± 13.1
Mean AV gradient (mm Hg)	C-XRT	47.9 ± 15.5	47 (41–57)	41 ± 9*	43.06 ± 13.67
	Control	45.9 ± 15.8	52 (46–65)	45 ± 13	40.87 ± 15.48

Values presented as n (%), mean (SD), or median (25-75th percentiles)

(*) indicates *p* valve < 0.05 for patients in the radiation group (C-XRT) compared to the control group

*BMI* Body mass index, *STS* Surgical Thoracic Society risk score, *LVEF* Left ventricular ejection fraction, *AV* Aortic valve, *NR* Not reported

### Risk of bias of the included studies

The included studies were together at moderate risk of bias according to the ‘Newcastle-Ottawa Scale’ assessment tool. The study of Dijos et al. has a very small number of patients in the C-XRT group as compared to the control group, and have not been adjusted adequately to the control group population in terms of age and peri-operative risk score [16]. The study of Bouleti et al. also has a small but equal number of patients in the comparison groups with a fair adjustment of the confounding factors between the comparison groups [17]. The studies of Agrawal et al. and Gajanana et al. have good quality selection with comparable patient-cohorts that are adjusted adequately for the confounders [10, 18]. The summary of the quality assessment domains from the included studies is shown in Table 3.

**Table 3:** Risk of bias assessment

Study ID	Selection	Comparability	Outcomes	NOS score
Dijos et al [16]	**	–	**	4
Bouleti et al [17]	**	*	**	5
Gajanana et al [18]	***	**	***	8
Agrawal et al [10]	***	**	***	8

(*) Asterisks denote the quality of each domain; NOS- Newcastle-Ottawa Scale. Numbers of stars in good quality: 3 or 4 in selection, 1 or 2 in comparability, and 2 or 3 in outcomes. Numbers of stars in fair quality: 2 in selection, 1 or 2 in comparability, and 2 or 3 in outcomes. Numbers of stars in poor quality: 0 or 1 in selection, 0 in comparability, and 0 or 1 in outcomes

### All-cause mortality

**Fig. 2:**
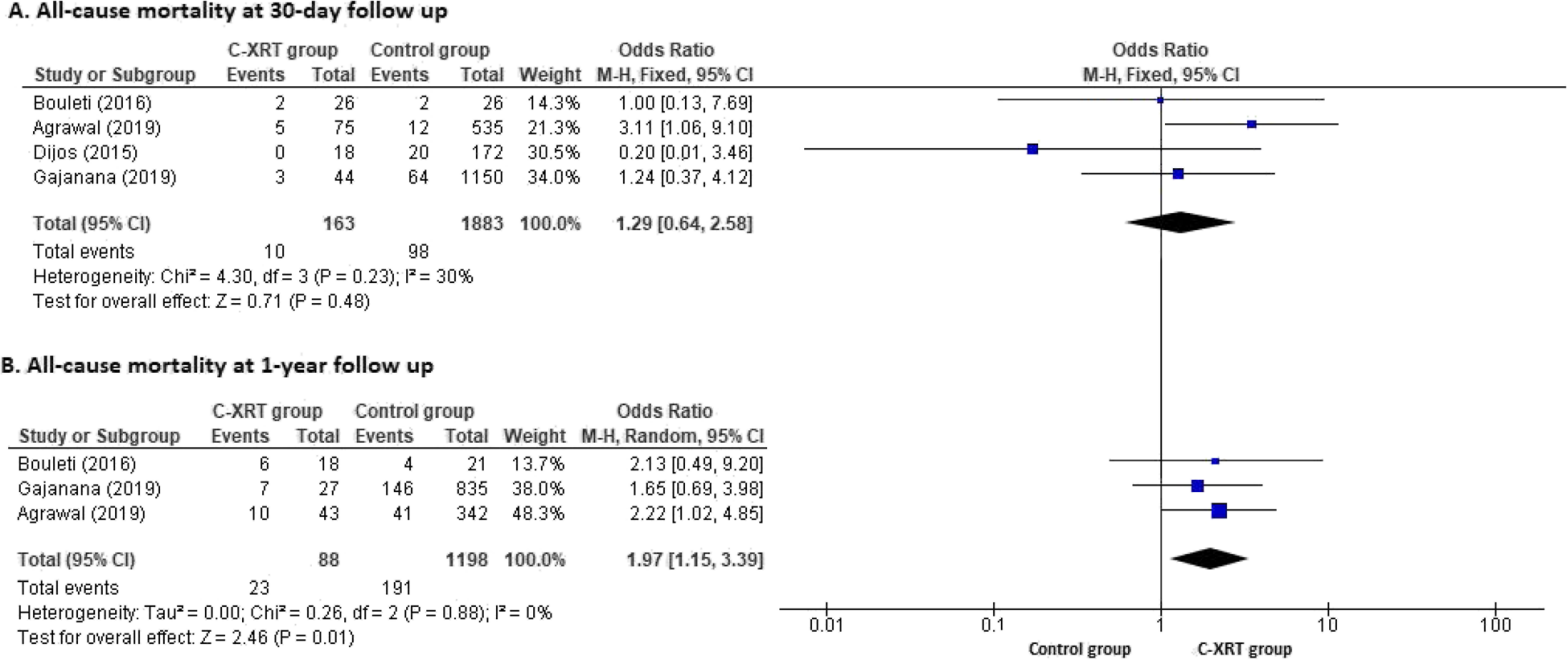
Forest plot for all-cause mortality. Forest plots with individual and summary estimates of the odds ratio (OR) with 95% confidence interval (CI) for the all-cause mortality at the 30-day follow up (**a**) and 1-year follow up (**b**). Squares and diamond size are proportional to the study weight

We analyzed the all-cause mortality at 30-day and 1-year follow-ups. The 30-day mortality outcome was reported in the four included studies and 1-year mortality was reported in the three included studies except Dijos et al. [16]. There was no statistically significant difference in the all-cause mortality at the 30-day follow-up when comparing the C-XRT group to the control group (OR 1.29, 95% CI 0.64 to 2.58, *p* = 0.48). However, the C-XRT group showed statistically significant higher all-cause mortality at 1-year follow-up compared to the control group (OR 1.97, CI 1.15 to 3.39, *p* = 0.01). The forest plots for the all-cause mortality at 30-day and 1-year follow-ups are shown in Fig. 2a and b, respectively.

### Safety outcomes (at 30-day follow-up)

#### Stroke (any)

**Fig. 3:**
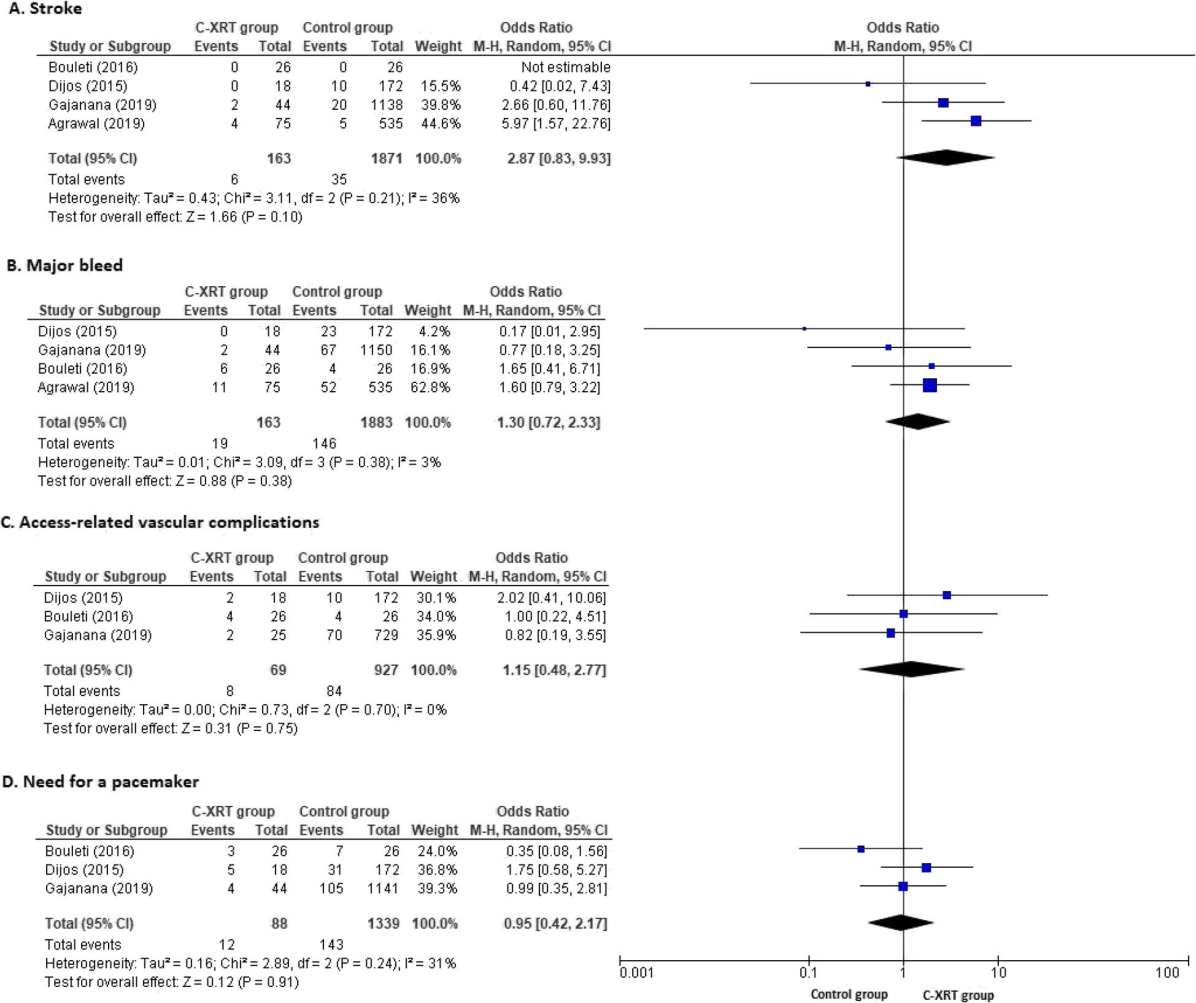
Forest plots for safety outcomes. Post-TAVR safety outcomes at 30-day follow-up in the C-XRT and control groups. Forest plots with individual and summary estimates of odds ratio (OR) with 95% confidence interval (CI) for stroke (**a**), major bleed (**b**), access-related vascular complications (**c**), and need for a pacemaker (**d**). Squares and diamond sizes are proportional to the study weight

This outcome was reported in all four included studies. According to pooled analysis, the C-XRT group suffers similar rates of strokes compared to the control group (OR 2.87, 95% CI 0.83 to 9.93, *p* = 0.10). The forest plot is shown in Fig. 3a.

#### Major bleed

This outcome was reported in all four included studies. There was no statistically significant difference in the major bleeding events between the comparison groups (OR 1.30, CI 0.72 to 2.33, *p* = 0.38). The forest plot is shown in Fig. 3b.

#### Access-related vascular complications

This outcome was reported in three included studies except for Agrawal et al. because it was not reported [10]. There was no statistically significant difference in access-related vascular complications in between the comparison groups (OR 1.15, CI 0.48 to 2.77, *p* = 0.75). The forest plot is shown in Fig. 3c.

#### Need for a pacemaker

This outcome was reported in all four included studies but we included data from three studies except for Agrawal et al. because it did not report the pacemaker implantation outcome at the 30-day follow-up [10]. According to pooled analysis, there was no statistically significant difference in the need for a pacemaker between the comparison groups (OR 0.95, CI 0.42 to 2.17, *p* = 0.91). The forest plot is shown in Fig. 3d.

### Efficacy outcomes

#### Left ventricular ejection fraction

**Fig. 4:**
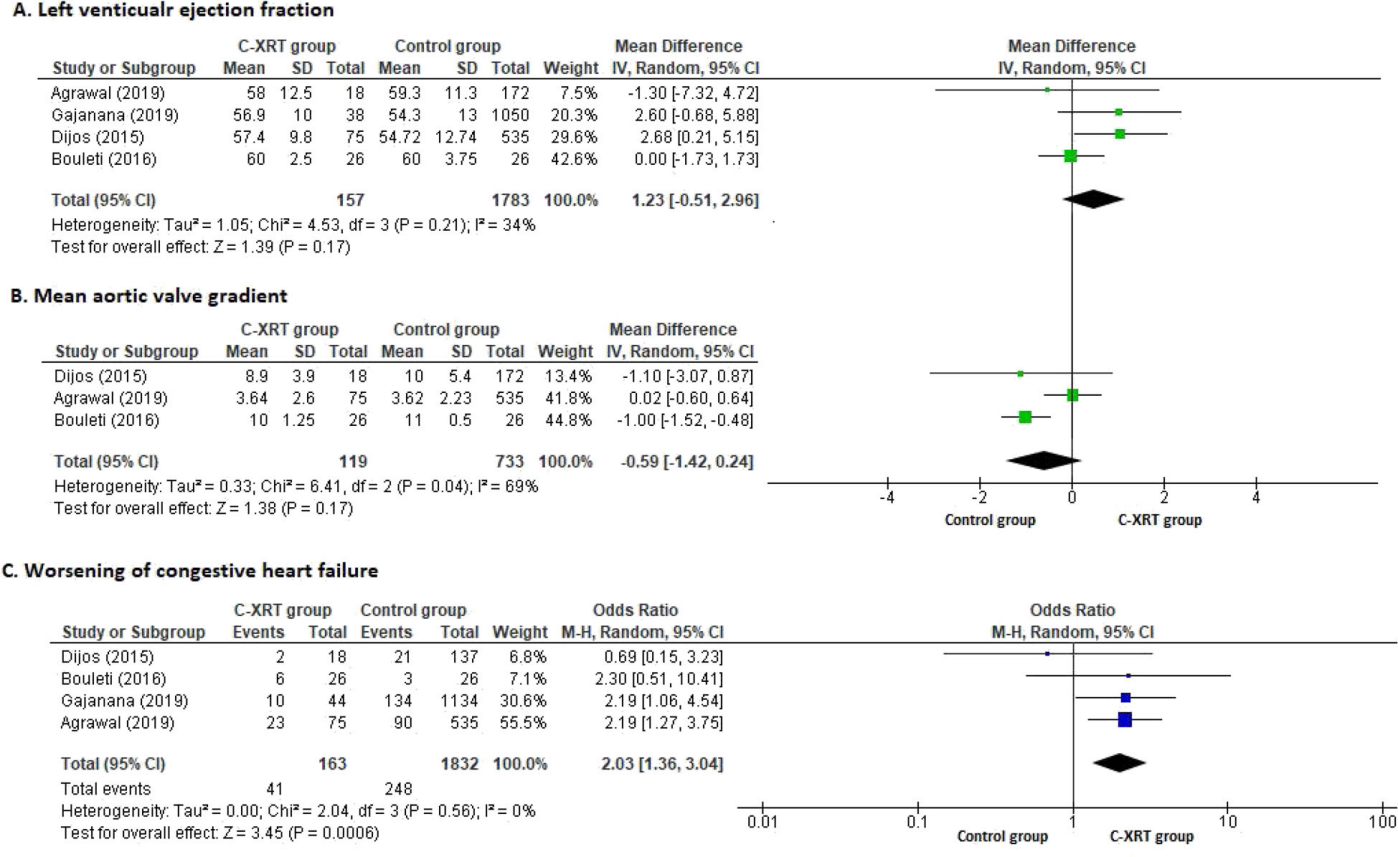
Forest plots for efficacy outcomes and worsening of heart failure. Post-TAVR efficacy outcomes and worsening of congestive heart failure (CHF) in the C-XRT and control groups. Forest plots with estimates of mean difference (MD) with 95% confidence interval (CI) for left ventricular ejection fraction (**a**), mean aortic valve gradient (**b**) and estimates of odds ratio (OR) with 95% CI for worsening of congestive heart failure (**c**). Square and diamond sizes are proportional to the study weight

This outcome was reported in all four included studies. There is no statistically significant difference between the comparison groups (OR 1.23, CI − 0.51 to 2.96, *p* = 0.17). The forest plot shown in Fig. 4a.

#### Mean aortic valve gradient

This outcome was reported in all four included studies but we analyzed data from three studies except for Gajanana et al. because it reported post-procedural mean aortic valve gradients as the difference in mean gradients with standard deviations [18]. We were unable to calculate appropriate data because co-variance was not reported by the author [18]. The pooled analysis showed no statistical significance between the comparison groups (OR -0.59, CI − 1.42 to 0.24, *p* = 0.17). The forest plot is shown in Fig. 4b.

### Post-procedural worsening of congestive heart failure

This outcome was reported in all four studies. The pooled analysis showed significantly higher rates of worsening congestive heart failure (CHF) in patients with prior C-XRT as compare to those without C-XRT (OR 2.03, CI 1.36 to 3.04, *p* = 0.0006). The forest plot is shown in Fig. 4c.

## Discussion

Radiotherapy is a preferred therapy to treat aggressive cancers including breast and lung cancers, Hodgkin’s lymphoma, and many other thoracic cancers. Unfortunately, radiation exposure leads to severe valvular disease including AS. Studies surrounding TAVR in cancer survivors with prior C-XRT have provided limited data. With this meta-analysis, we aim to add a piece of knowledge to help clinicians make better-informed decisions for this patient population. The central findings are: 1) TAVR seemed safe in cancer survivors with prior C-XRT, with similar short term all-cause mortality, safety and efficacy outcomes as in patients without prior C-XRT; 2) all-cause mortality at 1-year follow-up and post-procedural CHF exacerbation was higher in the C-XRT groups compared to the control groups.

Our analysis showed that the mortality rate 1-year after TAVR was nearly 2 times larger in the radiation group compared to the control group. One logical explanation of this finding could be the underlying comorbidities or the latent effects of cancer(s) and therapeutic regimens including radiation therapy. These results are similar to our previously published observational study, in which the multivariate analysis revealed radiation therapy as a potential factor for reduced long-term survival [10]. Landes et al. also reported similar mortality outcomes for TAVR in the cancer population, and higher mortality appeared to be driven by cancer progression [19]. A recent meta-analysis by Bendary and colleagues showed similar mortality outcomes for TAVR in patients with active cancer [26]. The Surgical Thoracic Society (STS) score generally possesses good predictive value for 30-day mortality in the setting of TAVR [27]. However, the duration of cancer, dose and duration of chemo-radiation therapy are not truly reflected in such risk assessment algorithms. Moreover, fragility is not uncommon among cancer survivors, and frail patients (defined by the Katz Index < 6) are at high risk of adverse early and late outcomes after TAVR [28]. Moving forward, we suggest applying additional risk assessment scores to better analyze the true benefits of TAVR in cancer survivors.

The post-procedural safety outcomes (stroke, major bleed, access-related vascular complications, and need for a pacemaker) measured at 30-day follow up were similar in both groups. In theory, TAVR seems to be a safer option as it overcomes technical aspects of performing open-heart surgery in patients with extensive chest radiation and mediastinal fibrosis. Better peri-procedural safety outcomes could be directly related to recent advancements in TAVR techniques. Patients with prior C-XRT had a higher incidence of atrial fibrillation, but with closer monitoring and anticoagulation use the incidence of stroke has declined. It is also likely that these patients have a higher incidence of atherosclerosis and aortic calcifications that are known to increase the stroke risk, especially with percutaneous vessel manipulation during TAVR [29–31]. With increasing use of the distal protective device during TAVR, the incidence of peri-procedural stroke has declined [32].

C-XRT is notorious to cause fibrosis of cardiac conduction pathways, which can be a substrate to induce arrhythmias. The routine electrocardiograms showed conduction defects in up to 75% of cancer survivors with prior C-XRT [33]. Watchful monitoring is desirable in these patients to detect and treat serious conduction abnormalities [34]. Our prior study also reported significantly higher post-procedural pacemaker implantation at subsequent follow-up after hospital discharge, signifying long term latent effects of radiation therapy [10]. Similarly, a recent study by Bendary et al. reported a higher need for post-procedural permanent pacemaker implantation at 30-day follow-up in patients with active cancer [26]. We were unable to analyze other safety outcomes like acute kidney injury, atrial fibrillation, myocardial infarction and so on, as they were not consistently reported in the included studies.

**Fig. 5:**
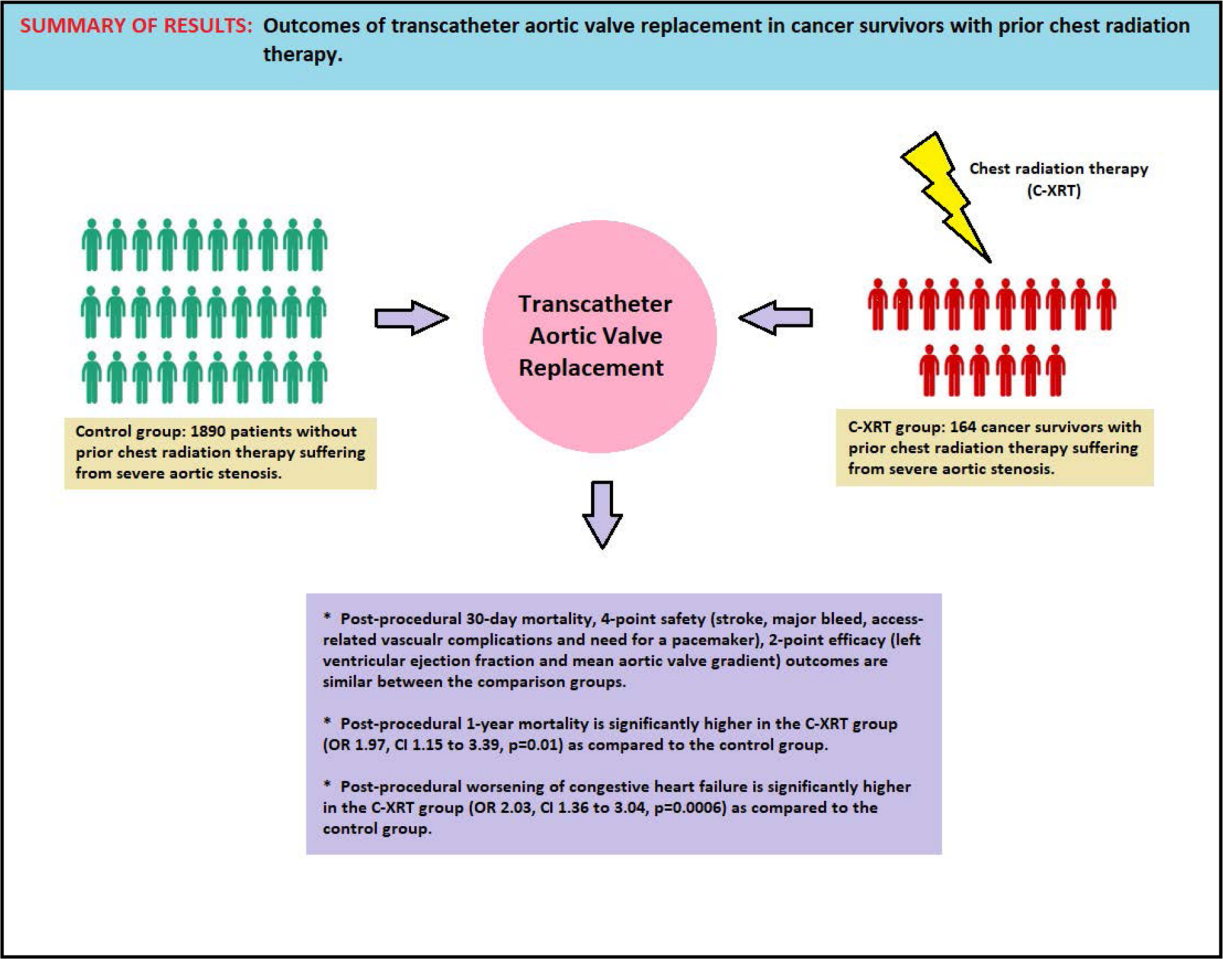
Schematic representation of the outcomes of transcatheter aortic valve replacement in cancer survivors with prior chest radiation therapy

In terms of morbidity, the post-procedural CHF exacerbation was nearly 2 times larger in the radiation group despite the pre- and post-procedure mean aortic valve gradients and LVEF being similar to the control group. One possible explanation is the development or worsening of diastolic dysfunction as radiation therapy has clearly shown to induce fibrosis resulting in impaired relaxation of cardiac myocytes [35, 36]. Additionally, our prior study reported a higher incidence of post-procedural anemia and blood transfusions in the radiation group and these factors were associated independently with the post-TAVR worsening of heart failure [10]. Another study by Durand et al. reported low aortic mean gradient, atrial dilation, post-procedural blood transfusion and pulmonary hypertension were associated with post-TAVR worsening of heart failure [37]. Moreover, the history of concomitant chemotherapy may have contributed to the higher incidence of heart failure exacerbations as many of these agents are known to cause cardiotoxicity [38]. These factors may account for worsening heart failure in this group despite intervention for severe valvular disease. Even when LVEF is normal, an abnormal strain is associated with higher mortality. Echocardiographic strain pattern imaging may help recognize those at risk who may benefit from timely intervention [39]. A recent study by Canada JM et al. reported impairment in the peak oxygen consumption (VO_2_), reduction in the diastolic functional reserve index (DFRI) and elevation of N-terminal pro-brain natriuretic peptide (NTproBNP) serum levels in the cancer survivors with prior C-XRT regardless of valvular dysfuntion [40]. Based on these data, we emphasize that the worsening of heart failure after TAVR in C-XRT patients is an important area for future studies to better understand the cardiopulmonary hemodynamic changes in the recepients of chest radiation therapy. The overview of the main findings of our study are summarized in the central illustration (Fig. 5**)**.

## Limitations

There are a few limitations of this study that warrant consideration. First, the incorporated studies are observational and, hence, are not without confounders and risk of bias. Despite being the best-obtained results, any conclusions drawn are hypothesis-generating and should be inferred cautiously. By all means, the randomized controlled trials comparing TAVR to standard medical therapy in such patients are critical to solve this clinical enigma; however, these types of studies are lacking, as shown by our meta-analysis. Second, our study population displays a widely heterogeneous and relatively smaller number of patients with different thoracic malignancies, variable therapies, and underlying comorbidities. Thus, it was arduous to stratify them based on types of malignancy. This would obligate access to an outsized patient database, which is not presently available. Third, we were unable to compare the outcomes of TAVR versus SAVR in cancer survivors with prior C-XRT due to lack of optimal number of available studies to analyze them meta-analytically. Finally, we cognize that data on long-term valve dysfunction are essential, but unfortunately, they were not conferred in the included studies.

## Conclusion

TAVR in cancer survivors with prior C-XRT have similar 30-day mortality, as compared to those without prior C-XRT. However, they have significantly higher 1-year mortality which may be due to the latent effects of cancer and therapeutic regimens including radiation therapy. Apart from a significantly higher rate of worsening CHF, TAVR is associated with similar safety and efficacy outcomes when compared to the control group. Applying additional risk assessment scores like frailty indices to the traditional preoperative risk assessment scores may help the cardiology team to determine the benefits of TAVR in the long run for these patients.

## Supplementary information

**Additional file 1: Supplemental Table.** Major Exclusions. **Figure S1.** Funnel plot of comparison: 30-day mortality. **Figure S2.** Funnel plot of comparison: 1 year mortality. **Figure S3**. Funnel plot of comparison: Stroke. **Figure S4**. Funnel plot of comparison: Major bleeding. **Figure S5**. Funnel plot of comparison: Access related vascular complications. **Figure S6.** Funnel plot of comparison: Need for a pacemaker. **Figure S7.** Funnel plot of comparison: Left ventricular ejection fraction. **Figure S8.** Funnel plot of comparison: Mean aortic valve gradient. **Figure S9.** Funnel plot of comparison: Post-procedural worsening of congestive heart failure.

## Data Availability

Authors can confirm that all relevant data are included in the article and/or its supplementary information files.

## Abbreviations

C-XRT: Chest Radiation Therapy
AS: Aortic Stenosis
TAVR: Transcatheter Aortic Valve Replacement
OR: Odds Ratio
CI: Confidence Interval
CHF: Congestive Heart Failure
SAVR: Surgical Aortic Valve Replacement
PRISMA: Preferred Reporting Items for Systemic Reviews and Meta-Analyses
STS: Society of Thoracic Surgery
LVEF: Left Ventricular Ejection Fraction
