# Supplementary material for: Outcomes after transcatheter aortic valve replacement in cancer survivors with prior chest radiation therapy: a systematic review and meta-analysis
Source: Cardiooncology. 2020 Jul 14;6:8. doi: 10.1186/s40959-020-00062-y (PMC7359474; doi:10.1186/s40959-020-00062-y)
Supplement: Supplementary file 1 — Additional file 1: Supplemental Table. Major Exclusions. Figure S1. Funnel plot of comparison: 30-day mortality. Figure S2. Funnel plot of comparison: 1 year mortality. Figure S3. Funnel plot of comparison: Stroke. Figure S4. Funnel plot of comparison: Major bleeding. Figure S5. Funnel plot of comparison: Access related vascular complications. Figure S6. Funnel plot of comparison: Need for a pacemaker. Figure S7. Funnel plot of comparison: Left ventricular ejection fraction. Figure S8. Funnel plot of comparison: Mean aortic valve gradient. Figure S9. Funnel plot of comparison: Post-procedural worsening of congestive heart failure. [file 40959_2020_62_MOESM1_ESM.docx]

# Supplemental Table - Major Exclusions

| **Trial** | **Journal** | **Reason for Exclusion** |
| --- | --- | --- |
| Landes et al. [19] | JACC Cardiovasc Interv | Lack of radiation exposure |
| Mangner et al. [34] | J Interv Cardiol | Lack of radiation exposure |
| Watanabe et al. [35] | Am J Cardiol | Lack of radiation exposure |
| Berkovitch et al. [36] | J Invasive Cardiol | Lack of radiation exposure |
| Biancari et al. [37] | Int J Cardiol | Lack of radiation exposure |
| Guhu et al. [38] | J Am Heart Assoc | Different study design. |
| Zhang et al. [39] | J Am Heart Assoc | Different study design |

**Figures**


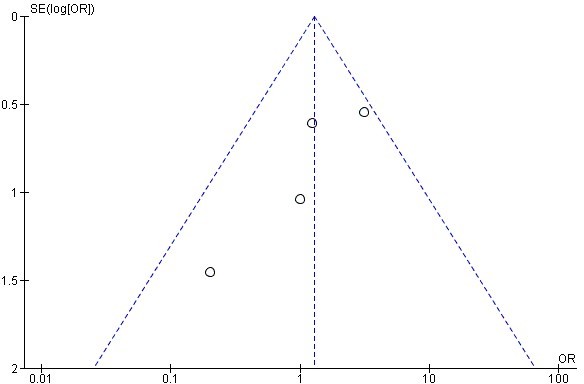
**Figure 1 (Analysis 1.1)**

Funnel plot of comparison: 30-day mortality.


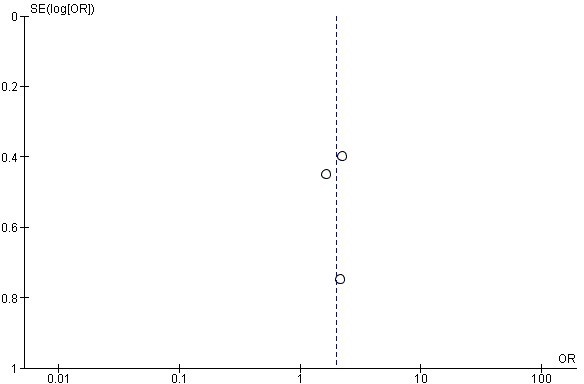


Funnel plot of comparison: 1 year mortality.


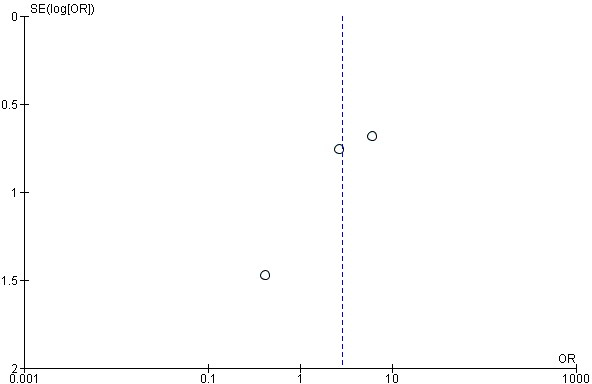


Funnel plot of comparison: Stroke.


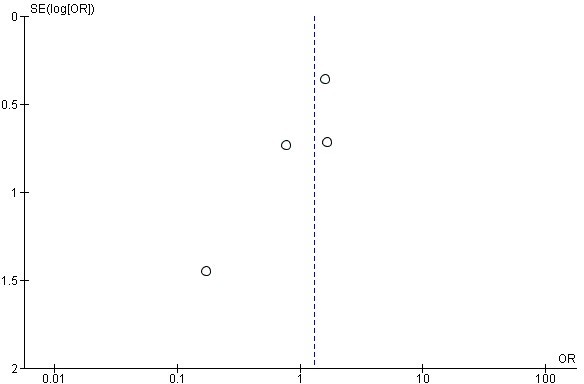


Funnel plot of comparison: Major bleeding.


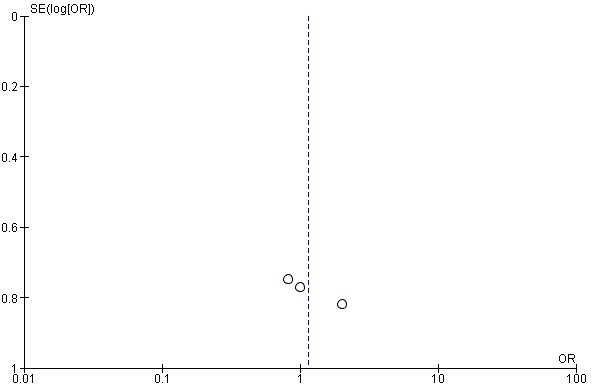


Funnel plot of comparison: Access related vascular complications.


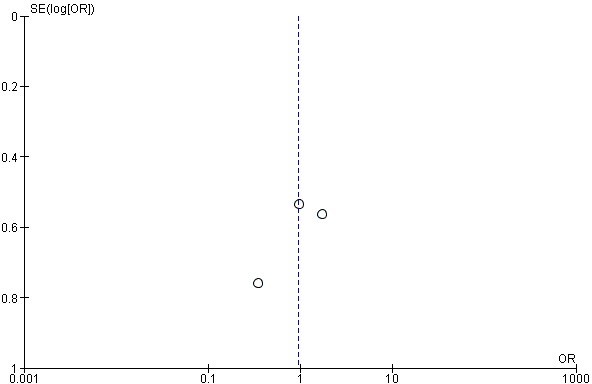


Funnel plot of comparison: Need for a pacemaker.


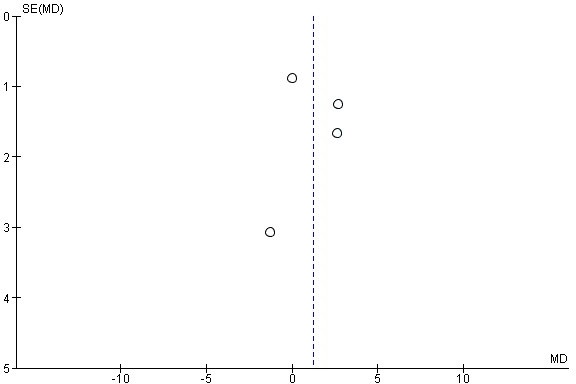


Funnel plot of comparison: Left ventricular ejection fraction.


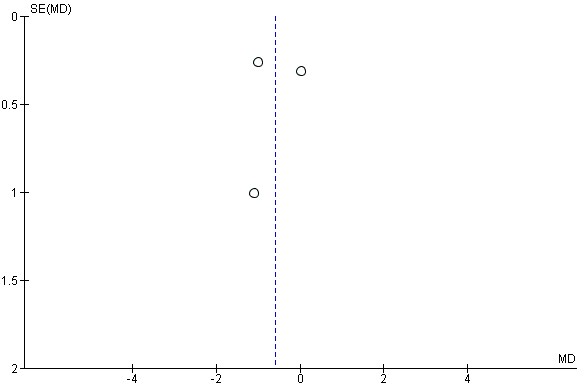


Funnel plot of comparison: Mean aortic valve gradient.


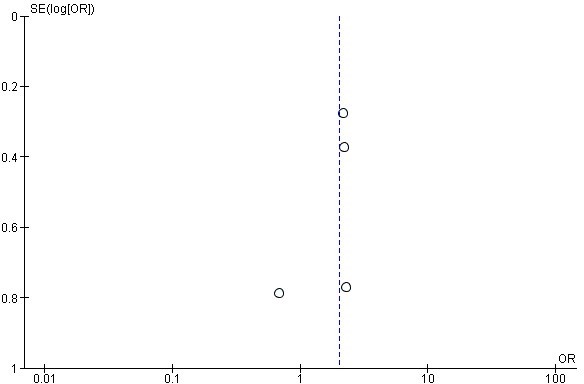


Funnel plot of comparison: Post-procedural worsening of congestive heart failure.
